# Inflammatory Response in the CNS: Friend or Foe?

**DOI:** 10.1007/s12035-016-0297-1

**Published:** 2016-11-26

**Authors:** Marta Sochocka, Breno Satler Diniz, Jerzy Leszek

**Affiliations:** 1 Hirszfeld Institute of Immunology and Experimental Therapy, Polish Academy of Sciences, Wroclaw, Poland; 2 Department of Psychiatry and Behavioral Sciences, and The Consortium on Aging, University of Texas Health Science Center at Houston, Houston, TX USA; 3 Department of Psychiatry, Wroclaw Medical University, Wybrzeże L. Pasteura 10, 50-367 Wroclaw, Poland

**Keywords:** Neuroinflammation, Immune response in the CNS, Microglia activation, Cytokines, miRNA, Neurodegeneration

## Abstract

Inflammatory reactions could be both beneficial and detrimental to the brain, depending on strengths of their activation in various stages of neurodegeneration. Mild activation of microglia and astrocytes usually reveals neuroprotective effects and ameliorates early symptoms of neurodegeneration; for instance, released cytokines help maintain synaptic plasticity and modulate neuronal excitability, and stimulated toll-like receptors (TLRs) promote neurogenesis and neurite outgrowth. However, strong activation of glial cells gives rise to cytokine overexpression/dysregulation, which accelerates neurodegeneration. Altered mutual regulation of p53 protein, a major tumor suppressor, and NF-κB, the major regulator of inflammation, seems to be crucial for the shift from beneficial to detrimental effects of neuroinflammatory reactions in neurodegeneration. Therapeutic intervention in the p53-NF-κB axis and modulation of TLR activity are future challenges to cope with neurodegeneration.

## Introduction

In the central nervous system (CNS), degenerative processes are characterized by morphological, anatomical, and functional changes that lead to early, chronic, and progressive neuronal loss. Chronic neurodegenerative diseases are defined as hereditary, sporadic, and protein misfolding diseases, which are usually characterized also by the decline of cognitive functions, particularly learning and memory. These include Alzheimer’s disease (AD) and other dementias, transmissible spongiform encephalopathies (TSEs), amyotrophic lateral sclerosis (ALS), Parkinson’s disease (PD), Huntington’s disease (HD), and prion diseases. The causes associated with neuronal degeneration remain poorly understood. Generally known risk factors for most neurodegenerative diseases are genetic polymorphisms and advanced age. The prevailing hypothesis is that the protein aggregates or seeds (α-synuclein, amyloid beta (Aβ), lipofuscin, tau protein) trigger a cascade of events leading to neurodegeneration and neuronal apoptosis [1–3]. Several other mechanisms may be involved in the pathogenesis of neurodegenerative disorders, including chronic inflammation, vascular factors, oxidative stress, and reduced availability of trophic factors in the brain.

Regulation of immuno-inflammatory control is one of the relevant processes involved in the pathogenesis of neurodegenerative disorders. Innate and adaptive immune response in the brain are tightly controlled in relation with the periphery. Immune activation in the CNS always involves microglia and astrocytes, which, in non-pathological conditions, contributes in the regulation of homeostasis of the brain tissue. Endothelia cells and perivascular macrophages are also important to the interpretation and propagation of inflammatory signals within the CNS [4]. In the CNS, microglia always scan the microenvironment by producing factors that influence adjacent astrocytes and neurons, particularly in response to infection or neuronal cell injury. This leads to the activation of an inflammatory response that further engages a transient, self-limiting response through the immune system and initiates tissue repair. Under pathological conditions, when the normal resolution mechanisms failed, there is an abnormal activation and production of inflammatory factors, leading to chronic neuroinflammatory state and progression of neurodegenerative changes.

Chronic neuroinflammation is observed at relatively early stages of neurodegenerative disease. The mentioned neurodegenerative factors impact on glial function by overactivation of both microglia and astrocytes triggering production and releasing large amounts of pro-inflammatory cytokines and reactive oxygen and nitrogen species (ROS, RNS). Chronic activation of microglia is linked to the degradation of protein, the dysfunction of mitochondria, and the defects of axonal transport and apoptosis, which have a detrimental effect on neuronal function and lead to cell death. Furthermore, neuroinflammation results in the subsequent infiltration of immune cells from the periphery to the CNS across the blood brain barrier (BBB), which accelerates neuroinflammation and neurodegeneration [5].

In this review, we aim to address the role of microglia, astrocytes, and immune response in the CNS in the development of neurodegenerative disorders. The review will present the “two faces” of neuroinflammation, which can result in the restoration of brain homeostasis as well as initiation or/and acceleration of neurodegenerative processes.

### Inflammation, Inflammaging, and Neuroinflammation

Inflammation is a complex biological response of the body to cell and tissue damages caused by chemical (acids, alkali), physical (ionizing radiation, magnetic field, ultrasonic waves), and biological factors (viruses, bacteria, fungi, exotoxins, and endotoxins) [6]. The type and range of inflammatory response depend on the type and intensity of the irritant. In addition, the tissue and organ resistance is also important. The potency of the irritant and the time of its impact on tissue determine the type of inflammatory state, acute or chronic. Inflammation can be beneficial as an acute, transient immune response to harmful conditions such as tissue injury or an invading pathogen. The proper inflammatory reactions facilitate the repair, turnover, and adaptation of tissues. In addition, moderate inflammatory reaction leads to the inhibition of bleeding resulting from trauma and removal of necrotic tissues, exotoxins, and endotoxins with exudation. Inflammation is a multistage response. The reactions of the mobility of cells, humoral response, i.e., activation of inflammatory mediators present locally and in body fluids, and the hemostatic response are engaged. The proper inflammatory response is self-limiting and characterized by an advantage of processes of restoring homeostasis over the destructive processes [7]. However, acute inflammatory response to pathogen-associated molecular patterns (PAMPs) may be impaired during aging, leading to increased susceptibility to infection. If the activity of the stimulating factor is persistent in time and the mechanisms of the proper development of inflammation are dysregulated, the body still receives a signal of health hazard and switches from the acute to a chronic inflammatory state [7, 8]. As a result, this causes an imbalance in the immune system, thereby the inflammatory markers remain permanently and generally at low grade. Chronic inflammation consecutively leads to the tissue degeneration and development of autoimmune or circulatory system diseases, arthritis, cancers, and CNS disorders [9].

Aging is a complex process that depends on many environmental factors and genetic and epigenetic events occurring in the different types of cells and tissues throughout life. Moreover, the aging process is a chronic oxidative and inflammatory stress, leading to damage of cell components, including proteins, lipids, and DNA, and contributing to the age-related decline of physiological functions [10]. “Inflammaging,” referred to as systemic, chronic inflammation, by Franceschi and Salvioli and colleagues [11, 12], is also the dominant feature of body aging and most, if not all, age-related diseases [8]. Many epidemiological studies confirm that inflammaging is a strong risk factor of various diseases, including AD, and death in the elderly. Inflammaging is connected with the increased level of inflammatory markers such as C-reactive protein (CRP) or interleukin-6 (IL-6) and also associated with many age-related changes, e.g., in the body composition, in the production and use of energy, in the maintenance of metabolic homeostasis, and in the immune response in the brain.

There are several possible mechanisms of inflammaging. Firstly, the inflammaging processes may be caused by the endogenous host-derived cell debris (damage-associated molecular patterns (DAMPs), i.e., damaged organelles, cells, and macromolecules) that accumulate with age as a consequence of both increased production and impaired elimination [8]. Secondly, aging cells and various inflammatory factors (termed the senescence-associated secretory phenotype or SASP) which they produce may be the chronic inflammation stimulators. Cellular senescence is a response to various stress factors and damages. Aging cells accumulate in various tissues where they contribute to the development of many pathological changes, for example modifying the tissue microenvironment and altering the function of nearby normal or transformed cells. Visceral adipose tissue (VAT) is the main place of senescent cell accumulation and is also a source of pro-inflammatory cytokines such as IL-6 and TNF-α [13]. Moreover, an excess and changes in the distribution of visceral adipose tissue and the composition and functioning of the lipids have clinical consequences such as metabolic syndrome. Metabolic syndrome is related to insulin resistance and impaired glucose tolerance, which lead to type 2 diabetes, obesity, dyslipidemia, elevated blood pressure, and activation of the pro-thrombotic and pro-inflammatory processes that lead to atherosclerosis and chronic inflammation [14, 15]. Studies of the association of distinct abdominal adipose tissue with the cardiometabolic risk factors and metabolic syndrome showed that metabolic syndrome individuals had significantly lower adiponectin levels and significantly higher levels of resistin, leptin, TNF-α, IL-6, intercellular adhesion molecule (ICAM), monocyte chemotactic protein-1 (MCP-1), and oxLDL than the control group. The results confirmed that deep subcutaneous adipose tissue (dSAT) is associated with increased inflammation and oxidative stress, suggesting that dSAT is an important determinant of metabolic syndrome [16]. A variety of adipokines, particularly interleukins, are considered to be associated with inflammatory processes that can lead to dementia and cognitive impairment. It is postulated that adipokines as biomarkers may enhance understanding of late-onset dementia risk over the life course, as well as the clinical progression of prodromal and manifest dementias [17]. Increasing evidence and clinical and epidemiological studies suggest an association between metabolic syndrome and type 2 diabetes and AD [18, 19]. It is indicated that diabetic patients have increased risk of developing AD and AD brains exhibit defective insulin signaling [20]. Thirdly, inflammaging may be caused by hyperactivity of the blood coagulation that increases the risk of thrombosis in the elderly. And finally, the reason for the development of inflammaging is the aging immune system (immunosenescence). Immunosenescence involves age-related remodeling changes in the organization of lymphoid organs and functions of immune cells, which have been associated with reduction of the degree of adaptive immunity and hyperactivity of the innate immune response. Immunosenescence may result from exposure to different pathogens and antigens over a lifetime, intracellular changes in immune cells, and genetic predisposition. Chronic infections, such as *cytomegalovirus* (CMV), *human immunodeficiency virus* (HIV), and *Epstein-Barr virus* (EBV) are known to impair the immune parameters [21–24]. Decline in cell-mediated immunity may in turn cause the age-related increased incidence of Herpes zoster (*varicella zoster virus*, VZV) and its complications in the elderly which is a worldwide growing problem for patient, cares, healthcare systems, and employers [25].

**Fig. 1:**
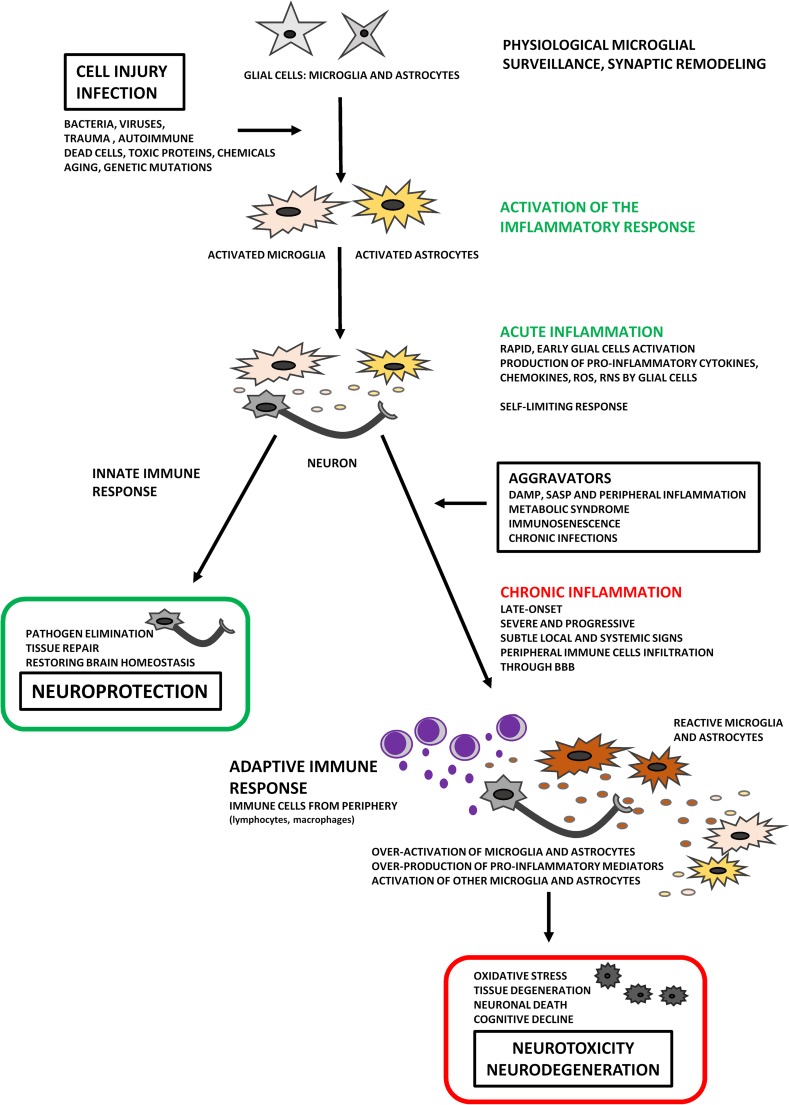
“Two faces” of neuroinflammation. Chronic inflammation is typically a prominent feature in the progressive nature of neurodegeneration. Neuroinflammation is an active process, which is dependent on well-orchestrated innate and adaptive immune responses, and the neuroinflammatory reactions may therefore be beneficial or detrimental, depending on their duration and strengths of activation

The term “neuroinflammation” means an inflammatory response originated in the CNS (brain and spinal cord) after injury by non-infectious or infection factors, with an accumulation of glial cells (microglia, astrocytes). The critical aspects in understanding neuroinflammation and its physiological, biochemical, and behavioral consequences are its context, course, and duration [4]. The active parts of the neuroinflammatory process take cytokines, chemokines, and complement and pattern-recognition receptors (PRR) that are produced and expressed by microglia and astrocytes [26, 27]. All the neuroinflammatory and regulatory processes within the CNS are generally initiated to prevent any disturbance of cell homeostasis. An acute inflammatory response in the CNS is caused by rapid and early activation of the glial cells as a response to different irritants (toxic proteins, infectious agents, stroke, depression, hypertension, diabetes, dementia, and other neurodegenerative disorders), which leads to repair of the damaged area of the brain. However, if harmful agents act persistent, an acute inflammatory state of the brain becomes chronic, and activation of glial cells is exaggerated, which leads to tissue degeneration. Moreover, chronic inflammation in the brain dysregulates mechanisms for clearing misfolded or damaged neuronal proteins resulting in tau-associated impairments of axonal integrity and transport, accumulation of amyloid precursor protein (APP), formation of paired helical filaments, and synaptic dysfunction. All these events precede and cause a prominent neurodegeneration and cognitive decline [27, 28]. Increased levels of inflammatory mediators, such as IL-1, IL-6, or TNF-α, are one of the biomarkers of human aging and closely associated with impaired mechanisms of ROS removal as well as leveling effects of their actions. Overgeneration of ROS leads to oxidative stress and induces NFκB expression, a key activator of inflammatory reactions. It is clear, therefore, that chronic inflammation in the CNS will occur frequently in people with age-related diseases [27]. Although the mechanisms that ultimately lead to neurodegeneration are different in each neurodegenerative disease (AD, PD, ALS, etc.), chronic inflammation is typically a prominent feature in the progressive nature of neurodegeneration. Thus, the resolution of inflammation is an active process, which is dependent on well-orchestrated innate and adaptive immune responses, and the neuroinflammatory reactions may therefore be beneficial or detrimental, depending on their duration and strengths of activation (Fig. 1) [29].

## Innate and Adaptive Immune Response in the CNS

The innate and adaptive immune systems actively participate in CNS surveillance, which is critical for the maintenance of CNS homeostasis and can facilitate the resolution of infections, degeneration, and tissue damage [30]. To understand neuroinflammation, it is important to distinguish innate and adaptive immune response in the CNS [5]. Innate immune reactions activated in the CNS lead to many essential modifications in the tissue microenvironment, e.g., changes in gene expression, which are normally repressed under physiological conditions and are only induced when cells are stressed, cellular differentiation, cellular composition and promotion of the recruitment of peripheral innate immune cells (macrophages, neutrophils) through BBB and adaptive immune cells (T cells and B cells). The main resident immune cells within the CNS are microglia, complemented also by CNS-derived macrophages from meninges, choroid plexus, and perivascular space, which provide innate immunity [31]. In non-pathological conditions, microglia scan the local microenvironment constantly and detect CNS damage. In this deactivation state, microglia release many immune (anti-inflammatory) and growth (neurotropic) factors that influence astrocytes and neurons. Cell injury or pathogen infection leads to microglial activation, morphological changes, and production of pro-inflammatory mediators. Thus, microglia are the earliest responders to any changes in the CNS [5, 32]. Developing an inflammatory response next stimulates the immune system (innate immune response), to eliminate stress stimulus. The initiation of an immune response may next involve the development of adaptive immunity. In the healthy brain, this early inflammatory response is self-limited, after the stimulus is terminated (phagocytosis of pathogens, abnormal protein deposits, debris or apoptotic cells) and described as beneficial and neuroprotective [33]. A recently characterized transient form of immune activation is euflammation, which can be induced by repeated subthreshold infectious challenges and causes innate immune alterations without overt neuroimmune activation. Thus, euflammation is associated with reduced inflammation and leads to neuroprotection [34, 35].

However, if inflammatory reactions are uncontrolled and chronic, it results in microglial overactivation (reactive microglia), which releases large amounts of inflammatory agents. This attracts other cells, microglia, and astrocytes. Innate inflammation is reported in AD, PD, ALS, and other neuropathologies [33]. Reactive microglia and astrocytes potentially cause injury to the BBB, which become more permeable for periphery immune cells, and neuronal impairment. The release of cytokines, chemokines, reactive oxygen species, and pro-inflammatory mediators by reactive glial cells leads to neurotoxicity and may accelerate neurodegeneration. Moreover, recruited peripheral immune cells (mainly lymphocytes) increase inflammatory response in the CNS by releasing more inflammatory mediators. Indeed, most CNS pathologies are often connected with abnormal microglial activation. An early phase of microglial activation is essential for the effective removal of toxic agents that could be detrimental for the brain. However, chronic microglial activation is connected with the overproduction of pro-inflammatory mediators which might override the beneficial effect of these cells [29].

It is worth noting that until now it was believed that neuroinflammatory response reflects systemic inflammation, which leads to the common view that entry of circulating immune cells to the CNS could only accelerate the parenchymal damage. González and Pacheco summarize the results of several studies showing that CD4(+) T cells infiltrate the CNS in many neurodegenerative disorders, in which their participation has a critical influence on the outcome of microglial activation and consequent neurodegeneration [36]. In fact, the CNS is constantly surveyed by circulating immune cells within the CSF, which entered into the brain through choroid plexus. The immune cell content of healthy CSF is estimated to consist of approximately 90% T cells, 5% B cells, 5% monocytes, and <1% dendritic cells [37]. In the physiological state, activated T cells, along with circulating and local innate immune cells, patrol the CNS and support brain plasticity, both in health and in response to CNS trauma. Schwartz and colleagues [29, 38, 39] demonstrated that the improvement of the CNS from acute damage is non-tissue autonomous and requires the involvement of circulating leukocytes, which are needed also for fighting off neurodegenerative conditions and which brought to appreciation the pivotal role of CNS-specific T cells in CNS maintenance and repair. Authors proposed a “protective autoimmunity theory” as an essential physiological mechanism for CNS protection, repair, and maintenance in both health and pathological diseases. This theory assumes a well-controlled generation and activation of CNS-specific T cells is a purposeful process, and only when it is dysregulated these cells become destructive. Yet, it is not confirmed whether protective autoimmunity is a more general phenomenon which occurs in tissues other than the CNS.

Importantly, inflammation is not only a pathological reaction that should be completely eliminated. The local inflammatory response and the innate and adaptive immune reactions are closely related with the etiology of each disease. Moreover, the inflammatory response involves a delicate balance between the innate and adaptive immune systems to deal with inflammatory stimuli [4, 29].

## Microglia and Astrocytes as Key Designers of the Resolution of Inflammation

### Microglia

Glial cells, described as non-excitable cells of the CNS, are a highly heterogeneous population, which initiate, participate, and regulate many important brain functions. Any discussion of neuroinflammation focused on the role of microglia and participation of astrocytes. Microglia, firstly described as brain-resident phagocytes, derive from the mesenchyme, in which myeloid stem cells give rise to cells, which migrate to the CNS and go through appropriate transformations [40, 41]. Currently, microglia are considered as the resident mononuclear phagocytes of the CNS, belonging to the glial system of non-neuronal cells. Microglia are broadly distributed throughout the brain, retina, optic nerve, and the spinal cord; however, they mainly reside in the hippocampus and gray matter and account for 5–20% of the total glial cell population within the CNS parenchyma. Microglia have an active role in immune surveillance. Over a decade ago, it was shown that under physiological conditions, microglia are not immunologically quiescent cells as previously believed and confirmed that they are the most dynamic CNS cells. Thus, microglia are now characterized as highly motile cells that contact synapses [42]. Microglia are highly specialized cells, which can either trigger neuroinflammatory pathways leading to gradual neurodegeneration or promote neuroprotection, downregulation of inflammation, and stimulation of neuron repair. Depending on the stage and context of any given lesion, one of these mechanisms prevails [43]. Based on many pathophysiologic studies, it is postulated that there are three different phenotypic states of microglia: (a) resting, ramified; (b) activated non-phagocytic (antigen-presenting cell (APC)-like) engaged in CNS inflammation; and (c) reactive, phagocytic, and present in areas of trauma or infection [44].

### Physiological Surveillance

Microglia are considered among the most versatile cells in the body, possessing the capacity to morphologically and functionally adapt to their ever-changing surroundings. Even in a steady state (microglia M0), the processes of microglia, “resting microglia” or rather “surveying microglia”, are highly dynamic and they perpetually scan the CNS. Recent investigations show fundamental roles for microglia in the control of neuronal proliferation and differentiation, as well as in the formation of synaptic connections [27, 45, 46]. Microglia are key regulators of synaptic remodeling during development and in the adult CNS via non-cell-autonomous mechanisms [47]. In the non-pathological brain, microglia mature and develop a ramified morphology characterized by motile processes that constantly monitor their immediate surrounding by extending and retracting their processes. Microglia are closely linked with neurons and determine their appropriate functioning (maturation and regeneration) by releasing several growth factors important for the proper development of the CNS. Microglia may play an important role in the remodeling of the brain by removing apoptotic neurons [48]. They were shown to be involved in the phagocytosis of synaptic elements during all stages of life. Microglia have a central role in the pruning of synapses by specifically engulfing the degenerating neurites of inappropriate connections. Stimulation of microglial phagocytosis with exosomes pointed out that exosomes may be a regulator of synapse elimination [49]. Exosomes are naturally occurring nanovesicles, which are implicated in the transfer of messenger RNA (mRNA), microRNAs (miRNA), lipids, and proteins between cells which lead to modifications of the functions of recipient cells. Bátiz et al. [50] present the molecules that could be expressed or secreted in exosomes under physiological or pathological conditions by CNS cells. Well-regulated communication between cells is essential to ensure brain homeostasis and plasticity. In healthy neurons, intercellular information transfer through exosomes acts as a unique mechanism for local and possibly systemic interneuronal transfer of information within functional brain networks [51]. Exosomes are actively involved in the communication between neuron and glial cells and between particular glial cells. It was shown that exosomes secreted by oligodendrocytes are endocytosed by neurons what improve neuronal metabolism and viability under conditions of cell stress (oxidative stress or lack of nutrients) [52]. Current studies confirmed that exosomes are present in the human CSF and may exert their function in brain sites located far from its secretion site. It is worth noticing that proteins related to the neuropathology of certain neurodegenerative diseases, like AD or PD, have been found in the exosomes from CSF samples [51]. Exosomes are also investigated to be involved in the processing of the APP which is associated with AD. These vehicles have been shown to contain full-length APP and several distinct proteolytically cleaved products of APP, including Aβ [53]. Moreover, Turola et al. report that microglia-derived exosomes can stimulate neuronal activity and participate to the propagation of inflammatory signals. They suggest that exosomes represent a secretory pathway for the inflammatory cytokine IL-β, and this process is activated by the ATP receptor P2X7 [54]. Thus, exosomes are considered as novel types of intercellular messengers that play important roles in cell function, disease, and immunomodulation [50, 55].

Microglia are also involved in the formation of learning-dependent synapses in the mature brain, as well as maturation and plasticity of excitatory synapses [42]. Wang et al. [56] investigated the constitutive role of microglia by depleting microglia from the mouse model of retina. Their results showed that sustained microglial depletion leads to the degeneration of photoreceptor synapses in the outer plexiform layer and causes a progressive functional deterioration in retinal light responses. They suggest that microglia are constitutively required for the maintenance of synaptic structure in the adult retina and for synaptic transmission underlying normal visual function. In the steady state, in the uninjured CNS, resting microglia contribute to neurogenesis processes, remyelination, and neuroprotection and also support tissue repair and are involved in the maintenance of brain homeostasis. The heterogeneity of microglia in serving housekeeping duties, sensing environmental signals, and organizing their (mostly) adequate responses to a disturbed CNS homeostasis is discussed by Gertig and Hanish [57].

### Inflammatory Activity

Microglia are very reactive cells; any changes in the CNS immediately lead to the activation, proliferation, and morphological changes of the cell structure [27, 58].In an early phase of acute neuroinflammatory response, the number of microglia increases immediately, and this is part of the microglial activation program [59]. As mentioned, microglia are the first line of defense against pathogens that invade and injure the CNS, contributing to both innate and adaptive immune responses locally. As phagocytes, microglia release cytotoxic factors and may act as APC. Microglia can be activated by a broad range of stimuli, including nerve injury, infection, ischemia, toxic insults, and trauma as well as different chemicals, cytokines, or proteins [60]. Moreover, C1q and C3b complement cascade proteins can activate innate immune response in microglia, thus inducing more vigorous response [43]. Among the spectrum of molecular targets, microglia sense and act on glycolipids, lipoproteins, peptides, nucleotides, Aβ, and other abnormally processed proteins, inflammatory cytokines, and neurons, the strongest inducers of microglial activation [61]. Luo and Chen [60] report that many studies emphasize the role of crosstalk between microglia and neurons in microglial activation. Healthy or injured neurons send different signals that determine neuroprotective or neurotoxic microglial activities. Activated microglia produce pro- and anti-inflammatory cytokines like TNF-α, IL-1β, IL-4, IL-6, IL-10, IL-12, IL-13, IL-15, IL-18, IFN-α, IFN-γ, TGF-β, M-CSF, and GM-CSF; chemokines (IL-8, Groα, IP-10, MIP-1α, MIP-1β); growth factors such as fibroblast growth factor (FGF), platelet-derived growth factor (PDGF), brain-derived neurotrophic factor (BDNF), and nerve growth factor (NGF); ROS; RNS; inflammatory markers (C-reactive protein, serum amyloid P); proteases (α-antitrypsin, α-antichemotrypsin); and complement system proteins [58, 61, 62]. Microglial activation is a complex process and may proceed in three different ways (microglia polarization), leading to (i) classical activation (M1), which is stimulated by IFN-γ, (ii) alternative phagocytic/neuroprotective activation (M2, now known as M2a with a subcategory M2b), which is stimulated by IL-4 and IL-13, and (iii) acquired deactivation (known as M2c), which is stimulated by TGF-β, IL-10, and apoptotic cells [63, 64]. M1 and M2 phenotypes, respectively, belong to the type (b) or (c) microglial states. Further, the factors which cause polarization to M1 or M2 reinforce the maintenance of that phenotype in a cycle-like manner [44]. Different antigenic markers characterize the microglial phenotypes, including HLA-DR, CD68, or ionized calcium-binding adaptor molecule-1 (IBA-1) as well as CD 14, CD 45, or ferritin [64]. The role of microglia is still debatable in terms of neuroprotection and neurodegeneration. Their dual activity is connected with the phenotype changing and interactions with other immune cells (astrocytes, T lymphocytes) [65].

In non-pathological states, microglia can support neurons by releasing neurotrophic factors and are capable of assisting in synaptic plasticity and structure remodeling [66]. Moreover, microglia play an important role in regulating neuronal network excitability. In the review of Ferrini and Koninck [67], the mechanisms by which BDNF, released from microglia, control neuronal excitability are described. They showed that microglia alter neuronal excitability by affecting synaptic inhibition mediated by *γ*-amino-butyric acid (GABA) and glycine (Gly) which activate ionic channels (GABA_A_R and GlyR) permeable to anions, like chloride (Cl^−^) and bicarbonate (HCO_3_^−^). Mild activation of microglia connected with the release of neurotrophic factors and cytokines, which translate environmental into molecular signals [68], has been shown to promote synaptic plasticity and promote neurons repair [69]. For example, certain basal levels of TNF-α are required for the development of normal cognition [70]. Steinmetz and Turrigiano [71] confirmed that glial-derived TNF-α is critical for maintaining synapses in a plastic state in which synaptic scaling can be expressed. Interestingly, the beneficial microglial state resembles an activated morphology and protein expression, but the function is distinct from a classic pro-inflammatory response. In general, microglial functions and activation are beneficial and necessary for a healthy CNS. If microglia become neurotoxic, it is always connected with the loss of the beneficial functions and/or a shift to a reactive phenotypic state. In this stage, the mechanism through which microglia are thought to cause neuron damage is through the excessive and inappropriate release of toxic factors [72].

The classical, M1, microglial activation pathway that initiates tissue defense mechanism is beneficial for the survival of the organisms and leads to the restoration of normal tissue homeostasis [63]. Many disease proteins and environmental toxicants trigger a toxic microglial response because they are misinterpreted as a pathogen M1 pathway which is connected with the activation of interferon regulatory factors (IRFs), especially IRF5, which in turn activates genes for pro-inflammatory cytokines IFN-γ, IL-1β, TNF-α, IL-6, IL-18, IL-12, and IL-23. This process is also related to the elevated level of NO, ROS, RNS, and chemokine and loss of phagocytic activity and support of defense-oriented Th1-type immune reactions [73]. Inflammatory agents regulate innate immune defense and modify synaptic function. To reduce the defense response and promote repair of the damage brain tissue, replacement of lost and damaged cells and restructuring of the damaged extracellular matrix are essential. The decrease in the activation of PRR and bystander injury caused by pro-inflammatory cytokines results from the reducing pathogen levels and the increasing catabolism of pro-inflammatory mediators. Moreover, during innate immune response in the brain tissue, invasion of monocytic cells from the periphery is also observed. Newly recruited macrophages phagocytose dead or dying immune cells then exit the tissue via the lymphatic system. This removal of the pro-inflammatory immune cells allows to restore tissue homeostasis [63]. However, strong activation of microglial cells can be associated with cytotoxicity. Overactivation of microglia, when they continually produce inflammatory mediators (chronic activation), can directly damage neurons and accelerate neurodegeneration [61]. Lull and Block suggest that many disease proteins and environmental toxicants trigger a toxic microglial response because they are misinterpreted as a pathogen [72, 74]. Longstanding microglial activation followed by sustained release of inflammatory mediators, which aid in enhanced nitrosative and oxidative stress, leads to chronic inflammation. The long-drawn release of pro-inflammatory mediators propels the inflammatory cycle by increased microglial activation and proliferation, thus stimulating enhanced release of pro-inflammatory agents [75]. Cytokines produced by microglia can stimulate another glial cells which next increase the pool of neurotoxic cytokines. Large amounts of pro-inflammatory cytokines, NO, ROS, and RNS lead to mitochondrial respiratory chain failure in glial cells and neurons [76]. Additionally, ROS may cause mutations in mitochondrial DNA (mtDNA), which in turn increase ROS production and deregulation of Ca(2+) homeostasis [43, 77]. Inflammatory factors secreted by microglia under the influence of Aβ may also increase the production of the Aβ. Impairment of intercellular communication leads to neurodegeneration and is connected with development of AD, PD, MS, ALS, Huntington’s disease, HIV dementia, and others [65, 72]. Moreover, extensive oxidative stress is linked with lipid peroxidation and oxidative modification of proteins [78]. Numerous studies confirm that the pro-inflammatory phenotype of microglia contributes to a reduction in the number of neurons, destabilizes synaptic connections, and impairs neurogenesis [79]. In fact, microglia present a tendency for a chronic pro-inflammatory response, rather than demonstrating a resolution of the innate immune response, as is common in the peripheral immune system. It is suggested that this tendency is a key factor driving progressive neuron damage, contributing to the chronic nature of neurodegenerative diseases [72]. As demonstrated, inhibition of microglial overactivation results in suppression of neurotoxic events and increases survival of neurons in early stages of neurodegeneration.

To stop the inflammatory phase of classically activated microglia, the change of macrophage activation state from pro-inflammatory gene profile to anti-inflammatory is essential. Microglia activated through the alternative, M2 pathway are characterized by increased level of anti-inflammatory cytokines, like IL-4, IL-10, IL-13, TGF-β, IGF, NGF, and BDNF, and increase in phagocytic activity without NO production. This phenotype assists Th2-type immune responses, resolves inflammation, and supports tissue repair and reconstruction [63, 73]. It is suggested that polarization to M2 microglia promotes remyelination. Recently, a new homeobox protein (msh-like homeobox-3 (Msx3))-dependent mechanism for driving microglia M2 polarization was described [80]. Increased phagocytic features allow for effective removal of Aβ deposits, which indicates the neuroprotective role of M2 microglia [26, 63]. The lack of an appropriate M2 response might be an important mechanism underlying neurodegeneration [81].

The third microglial activation state, associated with anti-inflammatory and repair activities, is an acquired deactivation (M2c phenotype). Both M2 phenotype and acquired deactivation downregulate innate immune response and present similar gene profiles. For that reason, many investigators include these two phenotypes into one category, but this is not justified. The explanation of the differences in acquired deactivation and alternative activation of microglia was previously shown by Colton [63]. In contrast to M2 activation, acquired deactivation is challenged by apoptotic cells, TGF-β and/or IL-10. Microglia are the main phagocytes engaged in the removal of apoptotic cells, and this mechanism is linked to suppression of pro-inflammatory cytokine production (immunosuppression of macrophage functions). TGF-β and IL-10 are released by several brain cell types including astrocytes and microglia. Additionally, an uptake of apoptotic cells increases the production of TGF-β and IL-10 by microglia. TGF-β and IL-10 have growth factor properties and promote survival of neurons and other cells through an activation of anti-apoptotic proteins, increasing tight junction at the BBB.

In the human brains, the classically, inflammatory activated microglia (M1) and an alternative, anti-inflammatory phenotype (M2) are present and are hybrids of these two phenotypes. It was shown that at the same time, different microglia can be at different stages of activation, differentiation, and function [64]. Currently, it is postulated that disturbances in the switching of microglial phenotypes may be one of the reasons for the development of chronic inflammation and neurodegenerative diseases. As a result, the relation of pro-inflammatory to anti-inflammatory phenotype is invalid, and it is known that microglial phenotype M1 is the biggest source of NO, ROS, RNS, and pro-inflammatory cytokines in the CNS that are disruptive to the adjacent neurons [82]. New approach to therapies in neurodegenerative diseases should also be based on to administer agents that inhibit the inflammatory stimulation of microglia or modulation of microglial activities by converting the inflammatory on anti-inflammatory phenotype [83]. Moreover, as suggested by Latta et al., evaluation of plasma proteins that are indicative of microglial immune profile (M1/M2) may allow for appropriate selection of patients for trials and immune therapy (personalized therapy) [84]. Microglial cell polarization may be regulated by many molecular signals, among which microRNAs have recently been identified. It is suggested that microRNA-155 (miR-155) regulates pro-inflammatory responses in both blood-derived and central nervous system (CNS)-resident myeloid cells [85]. Furthermore, microRNA-124 (miR-124) injection resulted in a significantly increased neuronal survival and a significantly increased number of M2-like polarized microglia/macrophages [86]. The role of miR-124 in the adaptation of microglia and macrophages to the CNS microenvironment and the influence of miR-155 and miR-124 on the polarization of macrophages are intensively discussed by Ponomarev et al. [87].

### Astrocytes

The second, most important glial cells are astrocytes. Astrocytes are ubiquitous and heterogeneous types of glial cells, which occupy 25 to 50% of the brain volume. Astrocytes are stellate cells, but their morphology differs depending on their development stage, subtype, and localization. Gray matter astrocytes are the protoplasmic ones, which exhibit short branches, whereas in the white matter, astrocytes exhibit long unbranched processes and are usually called fibrous astrocytes [88, 89]. Astrocytes are the only cells in the brain that contain the energy storage molecule glycogen, the largest energy reserve of the brain. They also contain a unique protein called glial fibrillary acidic protein (GFAP). It was presented that overexpression of GFAP can be lethal and is responsible for several neurodegenerative diseases, like Alexander disease [90, 91]. Astrocytes are multifunctional cells that control the brain homeostasis and are responsible for proper neuron functioning [58]. Their neuro-supportive role and participation in the formation and functioning of BBB are well documented. Astrocytes have an influence on pH, ion homeostasis and blood flow and regulate oxidative stress. Furthermore, these cells contribute to synaptogenesis, modulate neuronal conductivity, and regulate neural and synaptic plasticity [88, 92, 93]. Under physiological conditions, astrocytes can also metabolize Aβ. The receptor for advanced glycation endproducts (RAGE), expressed by astrocyte, binds Aβ, phagocytoses, and is taken up for lysosomal degradation in order to maintain Aβ homeostasis [89]. Astrocytes, like microglia, respond quickly on pathology within the CNS. They change the morphology, antigenicity, and function [58]. However, recent investigation suggests the dual role in either clearing and producing Aβ. Zhao et al. demonstrate that cytokines including TNF-α + IFN-γ and Aβ42 increase levels of endogenous beta-secretase 1 (BACE1), APP, and Aβ and stimulate amyloidogenic APP processing in astrocytes. These results suggest that mentioned factors promote astrocytic Aβ production, which means that activated astrocytes may represent significant sources of Aβ during neuroinflammation in AD. On the other hand, exposure to Aβ causes deleterious consequences on astrocyte functioning [94]. Thus, evidence suggests that astrocytes interact with neurons both chemically and physically, supporting their role as pivotal for higher brain functions (learning and memory). However, astroglial, as well as microglial, dysfunction following brain injury can alter mechanisms of synaptic plasticity and may be related to an increased risk for persistent memory deficits [69].

The interactions between astrocytes and microglia turn microglial inflammatory response. However, this mechanism could be impaired in inflammatory state where down-regulation of the astrocyte-suppressive function may lead to microglial overactivation and release large amounts of pro-inflammatory cytokines [65]. The numerous activities of astrocytes, similarly as microglia, following injury can either promote recovery or underlie the pathobiology of memory deficits [69]. Several studies investigate that the pathological changes of the astrocytes are associated with the occurrence of neurodegenerative diseases. Large amounts of astrocytes were found in the senile plaques in the brains of patients with AD and murine models, which is very characteristic of the disease progression and is described as reactive astrogliosis [27]. Astrocyte reactivity (astrogliosis) is characterized by three hallmarks, GFAP elevation, hypertrophy, and increased proliferation, and depends on interplay with activated microglia [26, 69]. Generally, astrocytes can be activated by various pathological factors, including Aβ, and pro-inflammatory cytokines such as IL-1β. Moreover, and the most important, is that astrocytes may be activated also by reactive microglia. Activation and inflammatory response of astrocytes is the response associated with the expression of many receptors for pro-inflammatory factors, including the receptors for cytokines IL-1β or TNF-α and chemokine. Astrocytes also produce ligands for TLRs. In response to this activation, astrocytic NF-κB is activated, and these cells release large amounts of pro-inflammatory cytokines, NO, and other neuroinflammatory agents, contributing to the increase in neuroinflammation in the brain and neuronal death. Astroglia-dependent toxicity was observed by Efremova et al. when immortalized murine astrocytes were stimulated with cytokine mix (TNF, IL-1) and the culture medium was transferred to human neurons [95]. The activation of NF-κB in astrocytes is also responsible in mediating the inflammatory process through the expression of adhesion molecules and chemokines which allow for the invasion by peripheral leukocytes, further fueling the inflammatory response [58].

#### Modulation of Microglial Activity

##### Receptors and Intracellular Signaling

Pathogens which penetrate BBB activate a mixed response of microglia characterized by enhanced phagocytosis and pro-inflammatory cytokine production, as well as adaptive activation of T cells. Thus, phagocytic activity of microglia may rescue neurons from degeneration and injury. Reactive microglia remove from the CNS not only pathogens but also damaged cells from neighboring tissues and maintain CNS homeostasis. CD200, expressed on the neuronal membrane, and its receptor CD200R present in the microglia are actively involved in phagocytosis. Interaction between these proteins determine the high threshold of microglial excitability, which allows for control of the inflammatory response in the CNS. The M2 activation pathway leads to increased CD200R expression under IL-4 stimulation. It was also shown, in the brains of the elderly and in AD patients, that the decrease in CD200 expression is age-related, which in turn increases the pro-inflammatory microglial activity or switch from M2 to M1 phenotype [60]. Microglial activation is also related to cytoskeletal rearrangements that alter the pattern of receptors on the cell surface. Microglial receptors include toll-like receptors (TLRs), which belong to the PRR that recognize PAMP and DAMP, nucleotide-binding domains, the leucine-rich repeat-containing receptors (NOD-like receptors (NLRs)), whose function is dependent on the multimolecular complexes termed “inflammasomes”, RAGE, Fc receptors, complement receptor 3, various scavenger receptors, C-type lectin receptor, mannose receptor MRC1, cytokine and chemokine receptors, receptors related to endocytosis (e.g., BIN1, PICALM, CD2AP) and lipid biology (e.g., CLU, ABCA7), several scavenger receptors, or receptors for several neurotransmitters [31, 62]. Moreover, TLR, SCARA1, CD36, CD14, α6β1integrin, and CD47 are important receptors for regulating microglial responses to Aβ. According to genome-wide association studies (GWAS), different gene variants of some of these receptors are associated with an increased risk of late-onset AD (LOAD) [96, 97].

Among PRR in CNS, membrane-bound TLRs, which sense extracellular or endosomally located signals, and NLRs, located within the cytoplasm and sense intracellular signals, are the key innate immune receptors expressed by microglia, macrophages, and astrocytes. NLRs are a part of the multiprotein complex called inflammasomes. Inflammasomes generally have three main components: a cytosolic PRR (which is a member of the NLR family of protein or pyrin and the HIN domain-containing family of proteins (PYHIN)), the enzyme caspase 1, and an adaptor protein that facilitates an interaction between the two [31]. This cytosolic platform enables the activation of caspase 1 which leads to the cleavage and release of pro-inflammatory cytokines. Inflammasomes are essential protein complexes that direct the innate immune system’s responses and apoptotic response in the human brain to pathogenic and non-pathogenic stimuli [98]. De Vasconcelos et al. present recent advances in the role of inflammasomes in regulated cell death signaling [99]. Indeed, initiation of the activation of inflammasomes in astrocytes and microglia leads to release in inflammatory factors, IL-1β and IL-18, which next activate more astrocytes and microglia and cause secretion of more inflammatory molecules. Inflammasomes are chiefly known for their roles in maturation and secretion of IL-1β and IL18. These molecules are responsible for the elevation of amyloidogenesis and neurofibrillary tangles (NFTs) in neurons and the recruitment of another immune cells (monocytes, lymphocytes) from the periphery, which are the source of even more pro-inflammatory factors. This feedback loop creates and propels neuroinflammation that leads to AD, PD, and other neurodegenerative disorders [100]. Many different types of stimuli may be the inflammasome’s activators, e.g., viruses, bacteria, fungi, protozoa, microbial proteins, crystalline urea, RNA, Alum, ATP, potassium efflux, Aβ, fatty acids, and degraded mitochondrial DNA [100]. In AD pathogenesis, it is postulated that activation of the NLRP3 inflammasome in microglia by Aβ may promote disease progression [98, 101]. Thus, NLRP3 is suspected to be a critical determinant of the development of low-grade sterile inflammatory responses during aging [102].

Positron emission tomography showed that microglial activation correlates with AD progression [103–105]. Aβ plays a pivotal role in the progression of AD through its neurotoxic and inflammatory effects. Aβ binds to microglia through receptor-mediated phagocytosis and degradation. Binding of Aβ to microglial membrane receptors appears to be a critical step. Activated microglia exert neuroprotection mediated through Aβ phagocytosis in the early stage, whereas, as the disease progresses, they fail in Aβ clearance and exert detrimental effects, including neuroinflammation and neurodegeneration [106–108]. Receptors expressed on microglia alone or with their co-receptors play complementary and non-redundant roles in the interaction with Aβ in AD. Pathogenic Aβ aggregate-activated microglia release various neurotoxic inflammatory mediators in classical M1 inflammatory activation [108]. Microglia express pattern recognition receptors, such as CD14 and especially TLRs, which were originally discovered based on their response to invading microorganisms [106]. TLRs are a family of pattern recognition receptors that are expressed by a variety of immune and non-immune cells [107, 108]. There are at least 13 distinct TLR family members known in mammals, of which the pathogen specificities of 10 (TLR 1–9 and 11) have been identified [108]. Recent studies have pointed out that immune stimulation targeting TLR9 could dramatically attenuate Aβ neurotoxicity and reduce Aβ levels in in vitro and in vivo AD models. Meanwhile, this reduction in amyloid is associated with cognitive improvement in AD mice [109–111]. Very important is that each TLR has a different ligand specificity that is extended through dimerization of the TLRs or additional co-receptors, such as CD14 for TLR4 and TLR2 [109, 112]. Recently, studies have provided evidence that CD14 and TLR2/TLR4 form a receptor complex, and together they participate in the inflammatory response induced by Aβ. It has been reported that CD14 binds fibrillary Aβ but not non-fibrillary Aβ. Neutralization with antibodies against CD14 and genetic deficiency of this receptor significantly reduced Aβ-induced microglial activation [112]. These results indicate that CD14 along with TLR4 can induce transcription factors such NF-kB nuclear translocation and consequently induce production of pro-inflammatory mediators in murine microglia and human peripheral blood monocytes [113]. Some studies cite crosstalk with NF-kB involving p53 as an example [113]. NF-kB and p53 can both be activated by many of the same stimuli with a common link frequently being DNA-damaging agents, which include ROS [114]. Besides CD14, there is also a direct interaction between TLR2 and the aggregated Aβ42. TLR2 deficiency reduces Aβ42-triggered inflammatory activation but enhances Aβ phagocytosis in cultured microglia and macrophages [113].

Recent studies focused on beclin 1 protein, which regulates autophagy, phagocytosis, and functioning of the receptors involved in this process in health and disease. Beclin 1 is involved in the degradation of proteins and immune defense. In mouse models of AD and PD, it has been shown that beclin 1 plays a key role in reducing amyloidosis and neurodegenerative processes. Beclin 1 deficiency results in reduced expression of the CD36 and TREM2 receptors that determine the proper process of phagocytosis. In AD brains, the expression of beclin 1 is decreased which is associated with ineffective phagocytosis and autophagy. Aβ deposits and the tau protein are not removed, which play a key role in AD pathogenesis [115–117]. One of the most important recent findings supports a role of immune dysfunction in AD, which is the connection between LOAD risk and TREM2 gene mutations [96]. TREM2, which belongs to the immunoglobulin (Ig) superfamily of receptors, and DAP-12, a type I transmembrane protein, form a receptor signaling complex on the cell surface of microglia, which triggers phagocytosis and the release of reactive oxygen species. TREM2, same as TLR4, can detect both a PAMP and a DAMP. TREM2 is able to bind gram-positive and gram-negative bacteria as well as anionic and zwitterionic lipids and interacts with other endogenous ligands on neurons, leading to the direct removal of damaged cells [118–120]. Anti-inflammatory properties of TREM 2 are well known. TREM2 reduces macrophage activation and inhibits cytokine production in response to TLR2 and TLR4 ligands [102]. Moreover, TREM2 is associated with increased phagocytosis and a promotion of a M2-like activation state of microglia, which is thought to have protective effects [121]. Mutations in TREM2, e.g., rare functional variant (R47H) [122, 123], cause impaired signaling by the TREM2-DAP12 pathway. The loss of the functionality of the complex leads to altered immune responses in phagocytosis, cytokine production, microglial proliferation, and survival, which in turn direct to the demyelination of neurons and development of dementia, increasing the risk for AD and other neurodegenerative disorders [118, 119]. Animal and human studies have indicated that TREM2 variants have been linked to an enhanced ability of microglia to clear Aβ and amyloid plaques. The loss of TREM2 functions is connected with Aβ-associated microgliosis and tau dysfunction [124–126]. Moreover, additional variants of TREM2, described by Colonna and Wang could be related to AD pathology. Based on these investigations, it is postulated that TREM2 variants may be the new key to deciphering Alzheimer’s disease pathogenesis [127].

##### Aging

Microglial activation has both detrimental and beneficial effects. Many studies with mouse model of AD suggests that early microglial activation is neuroprotective due to its Aβ clearance function, but as the disease progresses, pro-inflammatory cytokines downregulate genes involved in Aβ clearance, promoting Aβ accumulation [97]. Luo and Chen [60] showed the dual nature of microglia. Weather microglia have positive or negative effects on neuronal survival is context-dependent, but the aging has a great impact on microglial function and successive neurotoxicity. Thus, it was shown that the structure of aging microglia changes from a highly ramified morphology to spheroid formation with HLA-DR antigens, shortened and twisted cytoplasmic processes, and instances of partial or complete cytoplasmic fragmentation. This morphological alteration is described as “dystrophy” [59]. Moreover, the number of microglia increases and their layout becomes more irregular. Aging microglia function abnormally. They become less dynamic and more slowly respond to tissue injury [47]. The concept of “microglial aging” was proposed most recently. Microglial senescence is manifested by an altered inflammatory profile and switch from neuroprotective with production of anti-inflammatory mediators in young adult to neurotoxic with production of pro-inflammatory mediators in the aged brain upon activation [4, 60]. Importantly, chronic inflammation induces microglial aging from middle age. Senescent types of microglia respond incorrectly to stimuli and are driven by the emergence of increased intracellular ROS which activates the redox-sensitive transcription factors (including NFκB) and leads to mitochondrial DNA damage [78]. What is more, the NF-휅B signaling pathway may be activated by hypoxia and in turn induce microglial aging.

##### Timing

The timing of microglial activation is another determinant of their function, which decides microglia’s destructive or neuroprotective role in the CNS [60]. Hamelin et al. [128] investigated, in a prospective study using 18F-DPA-714 PET imaging, the microglial activation in early AD. They showed that microglial activation appears at the prodromal and possibly at the preclinical stage of AD and plays a protective role in the clinical progression of the disease at early stages. Importantly, the different dynamic profiles of microglial activation and their timing in the progression of the neurodegenerative process can be critical in identifying the correct therapeutic window to target microglial activation for disease modification [129].

##### The Role of Cytokines in Neuroinflammation

Cytokines play a key role in the induction and maintenance of neuroinflammation. They activate both microglia and astrocytes, but the duration of cytokine exposure is short and the effect is transient [4]. Activated astrocytes and microglia are in turn the main sources of cytokines in the CNS (Table 1). Numerous studies confirmed that the levels of classical pro-inflammatory cytokines such as IL-1, IL-6, IFN-γ, and TNF-α are elevated in chronic neurodegenerative diseases, especially in AD, which significantly contribute to the disease progression [138, 151, 153]. The correlation between AD prevalence and polymorphisms in IL-1, IL-6, TNF-α, and MIP-α genes was also demonstrated [154]. Additionally, the level of anti-inflammatory cytokines such as IL-4, IL-10, and IL-13 is generally reduced. Inflammatory state presents in the brains of AD patients and in transgenic mouse with cerebral amyloidosis, reaching a destructive size, which in turn increases the risk of transition from mild AD to dementia [26]. Thus, it is important that microglial and astrocyte actions are dependent on the nature of the activating stimulus. Smith et al. [138] summarize that microglial phagocytosis of invading pathogens is associated with their release of pro-inflammatory factors while clearance of apoptotic debris is associated with production of anti-inflammatory factors.

**Table 1:** Pro- and anti-inflammatory cytokines involved in the inflammatory response in the CNS

Cytokine	Role in the neuroinflammatory response	Literature
IL-1	Contributes to neuronal degeneration May induce apoptosis in neurons and glial cells Increases the level of APP and Aβ Increases the iNOS activity and NO production by astrocytes	[130–133]
IL-3	Neuroprotective effects against toxic activity of Aβ Released by peripheral leukocytes and microglia Microglial activator Anti-apoptotic activity mediated by Bcl-2 activation in neurons	[134–137]
IL-4	Induces microglia neuroprotective activity and neurogenesis Suppresses genes for pro-inflammatory cytokines IL-1 and TNF Switches microglia toward M2a response	[132, 138–140]
IL-6	Multifunctional cytokine Promotes astrogliosis and activation of microglia Contributes to neuronal degeneration May induce apoptosis in neurons and glial cells	[132, 133]
IL-8	Potentiates Aβ1–42-induced expression and production of pro-inflammatory cytokines in microglia May play a protective role in the AD pathogenesis	[134, 141]
IL-10	The main anti-inflammatory cytokine Plays an important role in neuronal homeostasis and cell survival Prevents overactivation and deficiency of the immune system Inhibitor of IL-1β, IL-6, and TNF-α secretion by microglia Controls the ROS and RNS production	[132, 142]
IL-12	Higher level in sera of EOAD (early-onset AD) patients Released by glial cells Regulator of immune responses	[143, 144]
IL-13	Suppresses genes for pro-inflammatory cytokines IL-1 and TNF Switches microglia toward M2a response	[132, 139]
IL-15	Marker of inflammation in the brain Microglial activator Unclear role in AD pathogenesis	[145, 146]
IL-18	Stimulates inflammatory factor production in the brain Increases tau phosphorylation and neurofibrillary tangle formation Accelerates aging processes and deteriorate brain cognitive functions	[147, 148]
IL-33	Nuclear alarmin (released after cell injury) Participates in gene silencing Amplifier of the innate immune response Induces glial cells to release inflammatory mediators causing either neuroprotective or neurotoxic effects (depending upon the concentrations) Stimulates microglial phagocytosis	[138, 149, 150]
TNF-α	Master regulator of the immune system Propagates inflammation Mediates the passage of periphery immune cells into the brain Dual activity—promotes neurodegeneration and apoptosis in neurons and glial cells, and also tissue regeneration Increases Aβ aggregation (as well as IFN-γ)	[76, 133, 151, 152]
IFN-γ	Important pro-inflammatory cytokine in the innate immune system Strong microglial and astrocyte activator Overexpression leads to decrease in Aβ deposits and infiltration of peripheral monocytes Both neuroprotective and neurodegenerative action depending on the concentration (low level induces microglial neuroprotective activity and neurogenesis) Antiviral activity	[94, 132, 151]

Generally, pro-inflammatory cytokines may directly contribute to neuronal degeneration, induce apoptosis in neurons and glial cells, increase BBB permeability, and promote trafficking of peripheral immune cells into the CNS, which contribute to damage of neurons. Additionally, these cytokines promote the increase in production of factors (ROS, NO) which are toxic for neurons [138, 155]. TNF is a strong pro-inflammatory stimulator for most cells of the immune system and the most important neuroinflammatory cytokine. In the case of CNS, TNF, released by activated microglia, may recruit periphery immune cells via the BBB into neuronal tissue, which is a critical step for the development of inflammatory diseases. Persistently elevated levels of TNF have been implicated in chronic inflammation and have been associated with neurodegenerative diseases. However, Fischer and colleagues confirmed earlier reports that TNF plays a region-specific and dual role in neurodegenerative diseases [76, 138, 151, 152]. They showed that the TNF receptor (TNFR) 1 is predominantly associated with neurodegeneration. Simultaneously, activation of TNFR2 signaling by TNC-scTNF(R2) promotes anti-apoptotic responses and leads to tissue regeneration and neuroprotection [76, 156]. Neuroprotective or neurodegenerative properties of TNF are also dependent on the concentration. The experiments with the use of primary cultures of astrocytes showed that the combination of pro-inflammatory cytokines such as TNF-α and IFN-γ increases the level of Aβ42 oligomers, APP and β-secretase. This in turn leads to an increase in the production of Aβ. These results indicate that activated astrocytes have a significant impact on the total volume of Aβ in AD during inflammation [94]. In the brain, IL-1, as an important regulator of the inflammatory cascade, is released primarily by activated microglial cells. It has been observed that IL-1 is the most important cytokine in the early stage of AD and its level is elevated in CSF and serum of AD patients [130, 134]. In vitro studies have demonstrated that IL-1 increases the level APP and Aβ, which leads to neuronal cell death [131]. Furthermore, L-1 may induce apoptosis, and this appears to be dependent on the presence or absence of additional cytokines (TNF-α and IFN-γ) and signaling molecules [138]. Pro-inflammatory cytokines, such as IL-1, can mediate in neuronal damage and death by stimulation of IL-6 production, induction in astrocyte iNOS activity, and release in nitric oxide (NO) and its derivative ONOO− [132]. The use of cytokine cocktail IL-1β + IFN-γ + TNF-α leads to the production of nitric oxide synthase (NOS-2) and a dangerously large amount of NO through activation of mitogen-activated kinases (MAPKs) by normal human astrocytes [157]. IL-33, a member of IL-1 family cytokines, is a pro-inflammatory cytokine, highly expressed in the CNS by endothelial cells and astrocytes but not by microglia or neurons. Microglia and astrocytes stimulated with IL-33 responded by proliferating and releasing inflammatory molecules such as TNF-α, IL-1β, and NO as well as the anti-inflammatory cytokine IL-10 [142]. Kempuraj et al. report that IL-33 mediates neurotoxic effects causing neuronal damage and neurodegeneration changes by releasing mentioned pro-inflammatory mediators (NO, TNF) and induction of CCL2 release from mouse astrocytes in vitro [158]. However, IL-33 and its receptor ST2 show both protective (physiologic) and anti-inflammatory activities depending upon the concentration and cell types/organ. IL-33 induces microglia and enhance phagocytosis, suggesting a protective role of IL-33 in neurodegenerative diseases [149]. IFN-*γ*, as TNF, has a pleiotropic nature. It possesses antiviral activity but also increases TNF activity and induces NO [132]. Interestingly, it was noted that acute but not chronic activation of certain types of immune responses, with short-term expression of IL-1, IL-6, and TNF, in the brain may be beneficial [58]. IL-8 exhibits the largest increase in expression of any inflammatory factor in human microglia incubated with amyloid-beta (Aβ1-42), and this increase is dose-dependent. Elevated levels of IL-8 in the CSF of AD patients have also been documented [134, 141]. Moreover, IL-8 has been reported to potentiate Aβ1-42-induced expression and production of a number of pro-inflammatory cytokines in cultured human microglia. Thus, IL-8 and its receptor CXCR2 contribute to chemotactic responses in AD. The results of Ryu et al. [159] evidence that upregulation of CXCR2 may be linked with microglial-mediated responses which in turn are correlated with neuronal damage in inflamed brain. However, recent studies demonstrate that IL-8 protects neurons possibly by paracrine or autocrine loop and regulates neuronal functions. Although IL-8 alone did not alter neuronal survival, it did inhibit Aβ-induced neuronal apoptosis and increase production of BDNF. Therefore, IL-8 may play a protective role in the AD pathogenesis [141]. Pro-inflammatory cytokine IL-12 is produced by microglia in response to cytokines, LPS, or a neurotropic virus [143]. Vom Berg et al. [144], using the APPPS1 AD mouse model, found increased production of the common interleukin-12 (IL-12) and IL-23 subunit p40 by microglia. Genetic ablation of the IL-12/IL-23 signaling molecule p40, p35, or p19 resulted in decreased cerebral amyloid load. Thus, they suggest that inhibition of the IL-12/IL-23 pathway may attenuate AD pathology and cognitive deficits.

Anti-inflammatory cytokines can suppress pro-inflammatory cytokine production and action, an effect that is critical to the concept of balance among pro- and anti-inflammatory cytokines. Il-10 as well as IL-4 has an anti-inflammatory activity and suppress the inflammation through inhibiting the secretion of IL-1β, IL-6, IL-8, IL-12, and TNF-α by microglia [132]. Moreover, IL-10 triggers microglia to M2c deactivation state. Zheng et al. [151] summarized current reports of IL-10 activity in the CNS. On the other hand, recent investigations showed that forced IL-10 expression in brains of APP transgenic mice leads to increased Aβ accumulation and worsening of behavioral deficits. Guillot-Sestier et al. [142] report that stimulation of microglia by recombinant IL-10 reduces Aβ phagocytosis, whereas IL-10 deficiency increases Aβ uptake by cultured microglia. It suggests that induction of a pro-inflammatory activation state endorses cerebral amyloid clearance. IL-4, as well as IL-13, is considered to be the strongest polarizing cytokine toward an M2a response [139, 140]. The neuroprotective effect of IL-4 might be related also to the inhibition of IFN-*γ* and the consequent decrease in the concentration of TNF-*α* and NO [132]. IL-3 could play a neuroprotective role in AD. According to recent literature, IL-3 level is reduced in the plasma of AD patients [134]. Zambrano et al. [135, 136] showed that IL-3 provides cellular protection against Aβ neurotoxicity in primary cortical neuronal cells. Moreover, they investigate that IL-3 induces an increase of the anti-apoptotic protein Bcl-2.

##### MicroRNAs and p53 as the Key Players in Neurodegeneration

The cause of AD has not been fully established; a close correlation between sporadic AD and the role played by p53 and microRNA is well documented in many publications [160–165]. The tumor suppressor and nuclear transcription factor p53 regulates major cellular functions, among them DNA synthesis and DNA repair, gene transcription, cell cycle, cellular senescence program, and cell death by apoptosis [160]. In post-mitotic neurons, p53 could be activated by various cell stressors, as hypoxia, oxidative stress, viral infections, metabolic stress, and trophic withdrawal, various insults which lead to DNA damage, oncogene activation, and excitotoxicity [160, 161]. According to severity of the stress signal, p53 protein helps in the cell adaptive response or, finally, triggers cell death program [162]. It is now clear that p53 plays an important role in neurodegeneration, and many studies reported neuronal cell death being associated with increased level of p53 in brain tissue cells [162, 166, 167]. Recently, the important function of p53 in the regulation of cellular metabolic homeostasis is revealed. By activation of its target transcription genes, p53 contributes to the regulation of glycolysis, glutaminolysis, oxidative phosphorylation, fatty acid oxidation, antioxidant activity, autophagy, and mitochondrial integrity [166, 168–170]. Lack of p53 or its abnormal folding affects neuronal function, leading to neuronal dysfunction [163, 171, 172]. P53 transactivates neuronal growth-associated protein-43 (GAP-43), a protein engaged in axonal growth and formation of new connections, and downregulation of GAP-43 expression is perceived as important molecular lesion that progresses with synaptic disconnections and neurodegeneration [173]. It is worth noticing that in cultures of fibroblast from AD subjects, exposure to low (nanomolar) concentrations of amyloid beta 1–40 peptide induced expression of aberrantly folded p53, and unfolded p53 could participate in the early pathogenesis of AD and would be a specific marker of the early stage of the disease [163, 173]. Together, data cited above accentuate the role of basal p53 level in the physiological regulation of metabolic, antioxidant, and regenerative processes. On the other hand, increased p53 expression induced by various chronic cellular stressors of moderate forth leads to significant changes in cellular metabolism, signaling, and expression of pro-oxidant target protein p53-inducible genes PIG3, PIG8, and ferredoxin reductase-FDRX [171], and these changes markedly contribute to progression of neurodegeneration.

##### MicroRNAs and a Crosstalk Between p53 and MicroRNA Network

MicroRNAs (miRNA) are single-stranded, small (19–23 nucleotides), endogenous, non-coding RNAs that regulate gene expression in eukaryotic cells by inducing translational arrest and degradation of messenger RNAs [164, 174]. MicroRNAs are proposed to allow organisms and cells to effectively deal with stress [175, 176]; in response to stress, cells adapt by altering their gene expression programs, upregulating a subset of mRNAs, which modulate the existing pool of mRNAs without any de novo synthesis, that is, by selectively translating certain mRNAs while halting translation of the rest [177]. Since miRNAs can also modulate the translation and/or stability of multiple targeted transcripts, it is assumed that miRNAs play an important regulatory role in coping with a spectrum of stresses, among them an oxidative stress, nutrient deprivation, DNA damage, or oncogenic stress [175, 177, 178]. The biological functions of miRNAs depend on the cellular context, i.e., on the differential expression of their target mRNAs in various cells which is preceded by specific action of transcription factors on gene expression. The p53 protein is a transcription factor which functions mainly by regulating expression of target genes; additionally, non-transcriptional functions of p53 are well documented [167–170, 179]. It regulates the expression not only of protein-coding genes but also of non-coding microRNAs, which act as mediators of p53 impact on gene expression. Interestingly, also the expression and activity of p53 itself are under the control of microRNAs [165]. In response to stress, p53 regulates microRNA synthesis and maturation, and the microRNAs participate in diverse cellular regulatory loops that modulate appropriate cellular adaptation [165, 180]. The transcription-independent modulation of microRNA biogenesis maturation and stability, which is carried out through p53 interaction with the processing complex (the Drosha complex), enables fine-tuning of cellular response to DNA damage and to other stresses of various origin [180, 181]. Likewise, transcriptionally inactive p53 mutants could interact with the Drosha complex leading to attenuation of several microRNA processing [181].

As p53 is a key player in the response to different types of cellular stress, its influence on several aspects of cell adaptations comprises also a metabolic shift in cells exposed to stress. The important executor of the p53 action on stressed cell is the microRNA network, and crosstalks between p53 and microRNA induction and processing are important in maintaining cellular homeostasis. Aberrant expression of the p53/microRNA axis leads to diseases, among them also to neurodegenerative processes. Future research on regulation of the p53/microRNA axis promises significant improvement of the repertoire of early diagnostic biomarkers and could open a new avenue for treatment of neurodegenerative disorders such AD.

##### Neuroinflammation: Friends or Foe?

The intrinsic inflammatory response of the CNS is a key player in the protection against CNS insults. The coordinate chain of events that initiate, modulate, and then lead to the resolution of inflammatory response help the CNS to fight against a myriad of local and systemic insults and maintain the brain health. However, in many instances, the delicate balance and control of the neuroinflammatory responses is lost and disease may arise. The imbalance of inflammatory responses in the CNS may be an initiating factor for many neurodegenerative diseases, i.e., Alzheimer’s disease. In other instances, the perpetuation of a chronic inflammatory response by activated microglia in response to the buildup of amyloid-β in the brain can lead to progressive neurodegenerative changes and neuronal death that ultimately lead to the clinical progression of dementia syndrome in AD. The relevance of neuroinflammation for maintenance of CNS health, as well as its being a player in several disease-initiating events and progression, makes it an interesting target for the development of novel treatment strategies for different CNS disorders.
